# Case report: Thymoma-associated GAD65 autoimmunity: a unifying mechanism for multi-organ injury involving nervous, endocrine, and renal systems

**DOI:** 10.3389/fimmu.2026.1790631

**Published:** 2026-03-12

**Authors:** Jiazhong Sun, Shiqi Sun, Zhiheng Sun, Qi Huang, Liman Luo

**Affiliations:** 1 Department of Endocrinology, Zhongnan Hospital of Wuhan University, Wuhan, China; 2 Department of Laboratory Medicine, The Sixth Hospital of Wuhan, Affiliated Hospital of Jianghan University, Wuhan, China; 3 School of Health and Nursing, Wuchang University of Technology, Wuhan, China

**Keywords:** epitope spreading, GAD65 autoantibodies, multi-organ autoimmunity, paraneoplastic syndrome, thymoma

## Abstract

Thymomas are well-established disruptors of central immune tolerance, yet their capacity to drive systemic autoimmunity via a single autoantigen remains incompletely characterized. We report a 59-year-old male who, following resection of a Masaoka stage IIb type B2 thymoma, developed sequential neurological, endocrine, and renal manifestations over 18 months. Clinical evaluation confirmed stiff-person syndrome (SPS) with axial muscle hypermetabolism on PET-CT, latent autoimmune diabetes (HbA1c 11%), subclinical hypothyroidism (TSH 15.28 μIU/mL), and distal renal tubular acidosis (dRTA) evidenced by severe hypokalemia (K^+^ 2.4 mmol/L) and inappropriately alkaline urine (pH 7.5). All three syndromes were associated with persistently high-titer glutamic acid decarboxylase 65 (GAD65) autoantibodies (>500 nmol/L in serum). Epitope mapping identified molecular mimicry between the GAD65 peptide ²^60^PEVKEK²^65^ and homologous sequences in renal H^+^-ATPase and thyroid peroxidase, providing a mechanistic basis for multi-organ cross-reactivity. Treatment with intravenous immunoglobulin (IVIG) led to a 53.6% reduction in serum and an 80% decline in cerebrospinal fluid GAD65 antibody levels, paralleling clinical improvement. This case illustrates how thymoma-induced loss of tolerance to GAD65 can trigger widespread autoimmune injury through conformational epitope spreading, positioning GAD65 as a paraneoplastic pan-autoantigen. High-titer GAD65 antibodies may serve as a biomarker for systemic involvement, supporting early multidisciplinary surveillance in thymoma patients.

## Introduction

Autoimmune diseases arise from a breakdown in self-tolerance, leading to immune-mediated injury across multiple organ systems—often orchestrated by autoantibodies directed against antigens expressed in diverse tissues (1). Among the neoplastic drivers of such systemic autoimmunity, thymomas hold a distinctive role. As tumors of thymic epithelial origin, they frequently impair central tolerance mechanisms, giving rise to a range of paraneoplastic syndromes (2). Up to 30–65% of patients with thymoma develop myasthenia gravis, while 5–15% present with pure red cell aplasia or hypogammaglobulinemia (3, 4).

Here, we describe a patient with a type B2 thymoma who developed high-titer glutamic acid decarboxylase 65 (GAD65) autoantibodies and a striking triad of autoimmune manifestations: stiff-person syndrome (SPS), distal renal tubular acidosis (dRTA), and latent autoimmune diabetes in adults (LADA) (5). Although GAD65 antibodies are classically linked to type 1 diabetes and certain neurological disorders, their association with renal tubular dysfunction remains rare. Emerging evidence suggests that structural features of GAD65—particularly conformational epitopes exposed under inflammatory conditions—may enable cross-reactivity with proteins in the central nervous system, pancreas, and kidney via molecular mimicry (6–8).

This case highlights three key aspects of thymoma-associated autoimmunity. First, B2 thymomas can profoundly disrupt immune homeostasis through mechanisms including AIRE downregulation, regulatory T-cell (Treg) impairment, and defective negative selection of autoreactive T cells (9, 10). Second, GAD65 may act as a pan-autoantigen: its expression in neurons, pancreatic β-cells, and renal intercalated cells provides a plausible anatomical basis for multi-organ targeting (11, 12). Third, autoimmune manifestations can evolve over an extended period—even after complete thymectomy—underscoring the long-lived nature of autoreactive lymphocyte clones once central tolerance is breached (2, 13). Notably, autoimmune manifestations in thymoma patients can precede the tumor diagnosis, as the insidious growth of the thymoma and the prolonged incubation period of autoreactive lymphocyte clones may lead to a delayed presentation of organ-specific injury.

Collectively, this report broadens the recognized spectrum of GAD65-mediated autoimmunity in the context of thymoma and reinforces the importance of systematic, long-term surveillance for extra-neurological and extra-endocrine involvement in affected patients (14).

## Case presentation

In December 2023, a 59-year-old man was evaluated for persistent hypokalemia (serum potassium 2.4 mmol/L; reference range 3.5–5.3) that failed to correct with oral potassium supplementation. Laboratory evaluation revealed metabolic acidosis with a serum bicarbonate of 9.7 mmol/L. The calculated anion gap was elevated at 23.3 mEq/L (Na^+^ 141, Cl^-^ 108 mmol/L), suggesting a mixed acid-base disorder. The concomitant finding of inappropriately alkaline urine (pH 7.5) in the face of systemic acidosis pointed to a distal renal tubular acidification defect, consistent with distal renal tubular acidosis (dRTA). A 24-hour urinary potassium excretion of 63.8 mmol confirmed renal potassium wasting. Over the preceding six months, the patient had developed progressive bilateral lower-limb weakness, which ultimately left him unable to walk. This neuromuscular deficit was subsequently attributed to severe hypokalemia secondary to dRTA. Genetic testing did not identify mutations associated with inherited distal renal tubular acidosis, and adrenal imaging ruled out hyperaldosteronism; on this basis, dRTA was diagnosed. In addition, laboratory evaluation revealed subclinical hypothyroidism (thyroid-stimulating hormone 15.3 μIU/mL; reference 0.25–5.8) and impaired glucose tolerance, with a 2-hour plasma glucose of 9.3 mmol/L during a standard oral glucose tolerance test. Serum for autoantibody testing, including GAD65, was not collected at this initial presentation.

**Figure 1:**
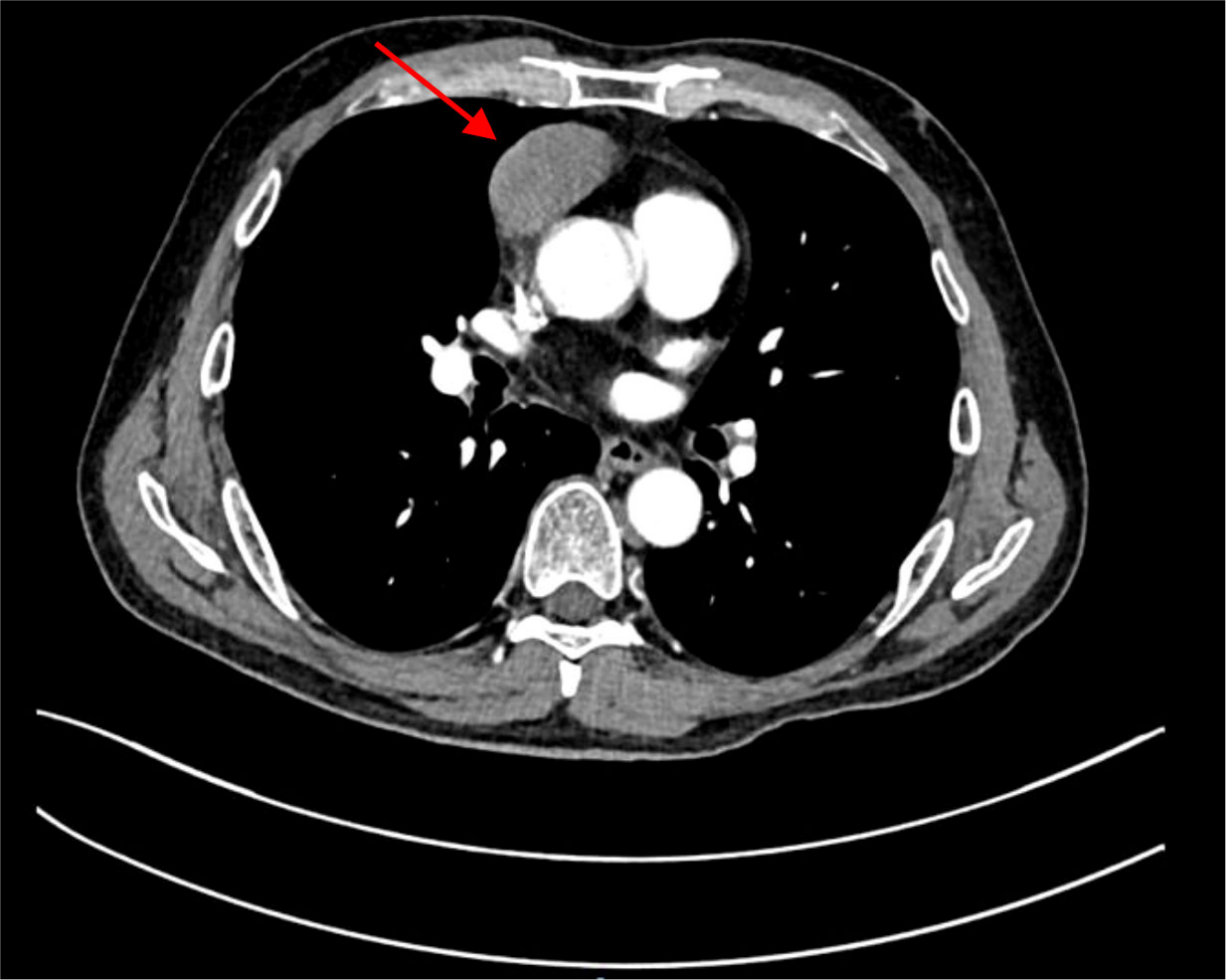
Contrast-enhanced chest CT scan showing an anterior mediastinal mass. A 5.0 × 4.3 × 2.5-cm well-circumscribed, heterogeneously enhancing mass is present in the anterior mediastinum (arrow), consistent with thymoma. The lesion abuts but does not invade adjacent great vessels.

**Figure 2:**
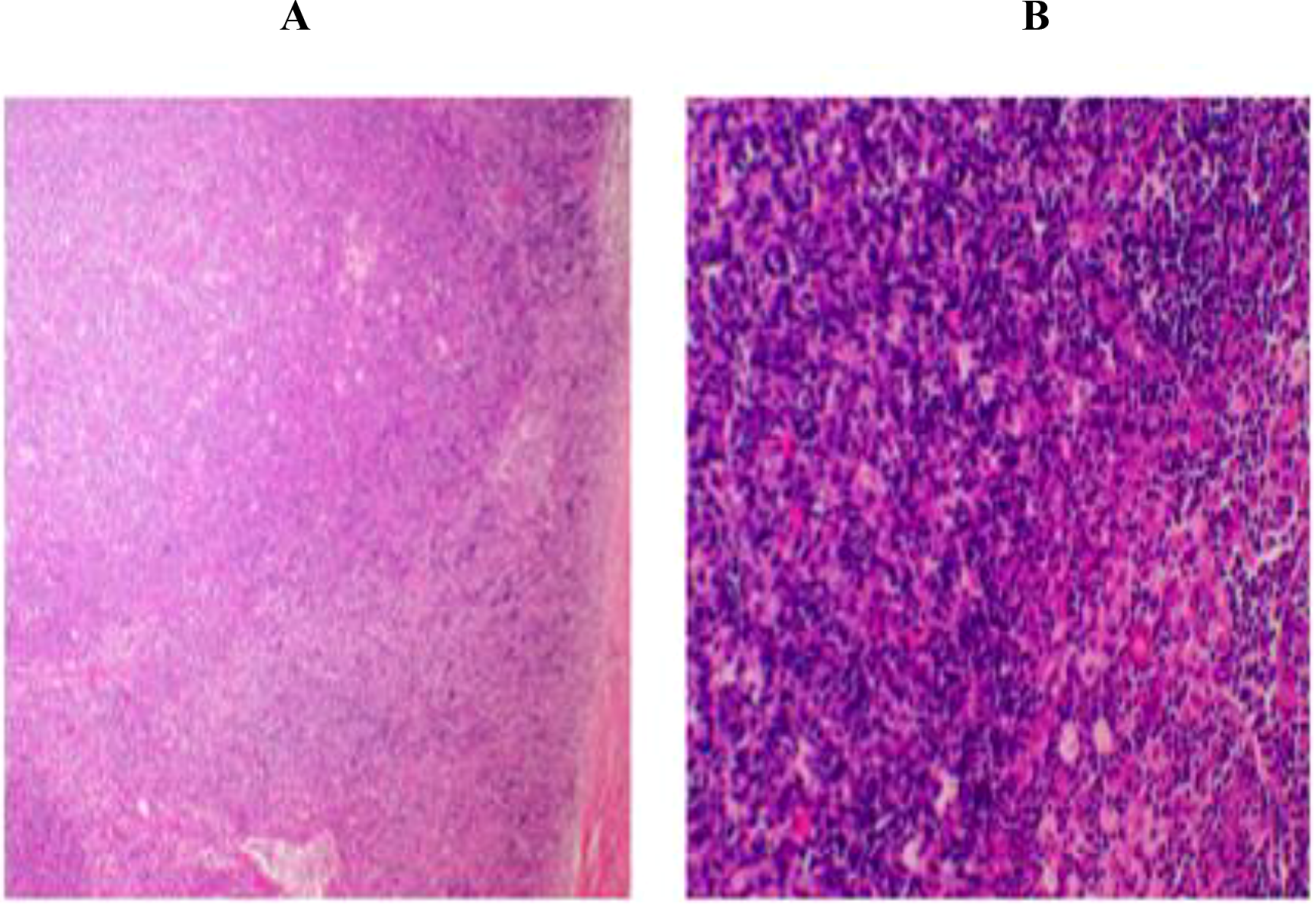
Histopathologic and immunohistochemical features of type B2 thymoma (Masaoka–Koga Stage IIb). **(A)** Hematoxylin and eosin staining shows lobular architecture with medullary differentiation (left) and a cortical-rich area with increased cellularity, nuclear pleomorphism, and prominent nucleoli (right; original magnification, ×200). **(B)** Immunohistochemical analysis reveals tumor cells positive for cytokeratin 14, cytokeratin 19, p63, and p40; scattered immature T cells are TdT-positive, and rare B cells are CD20-positive. The Ki-67 proliferation index is approximately 70%. Staining for CD117 and epithelial membrane antigen is negative, supporting a diagnosis of thymoma rather than thymic carcinoma.

A chest CT scan in January 2024 revealed a 5.0 × 4.3 × 2.5-cm anterior mediastinal mass (**Figure 1**). Thymectomy was performed, consisting of en bloc resection of the thymoma with preservation of surrounding thymic tissue; no total thymectomy was carried out. The histopathologic analysis showed type B2 thymoma (Masaoka stage IIb) with a Ki-67 labeling index of 70% (**Figure 2**). Immunohistochemical staining was positive for CD5, CK14, CK19, p63, p40, and TdT, with scattered CD20^+^ cells; CD117 and EMA were negative. Acetylcholine receptor antibodies were undetectable (<0.01 nmol/L). The patient received adjuvant radiotherapy (total dose 50 Gy in 25 fractions) and chemotherapy (4 cycles of cisplatin and etoposide).

**Figure 3:**
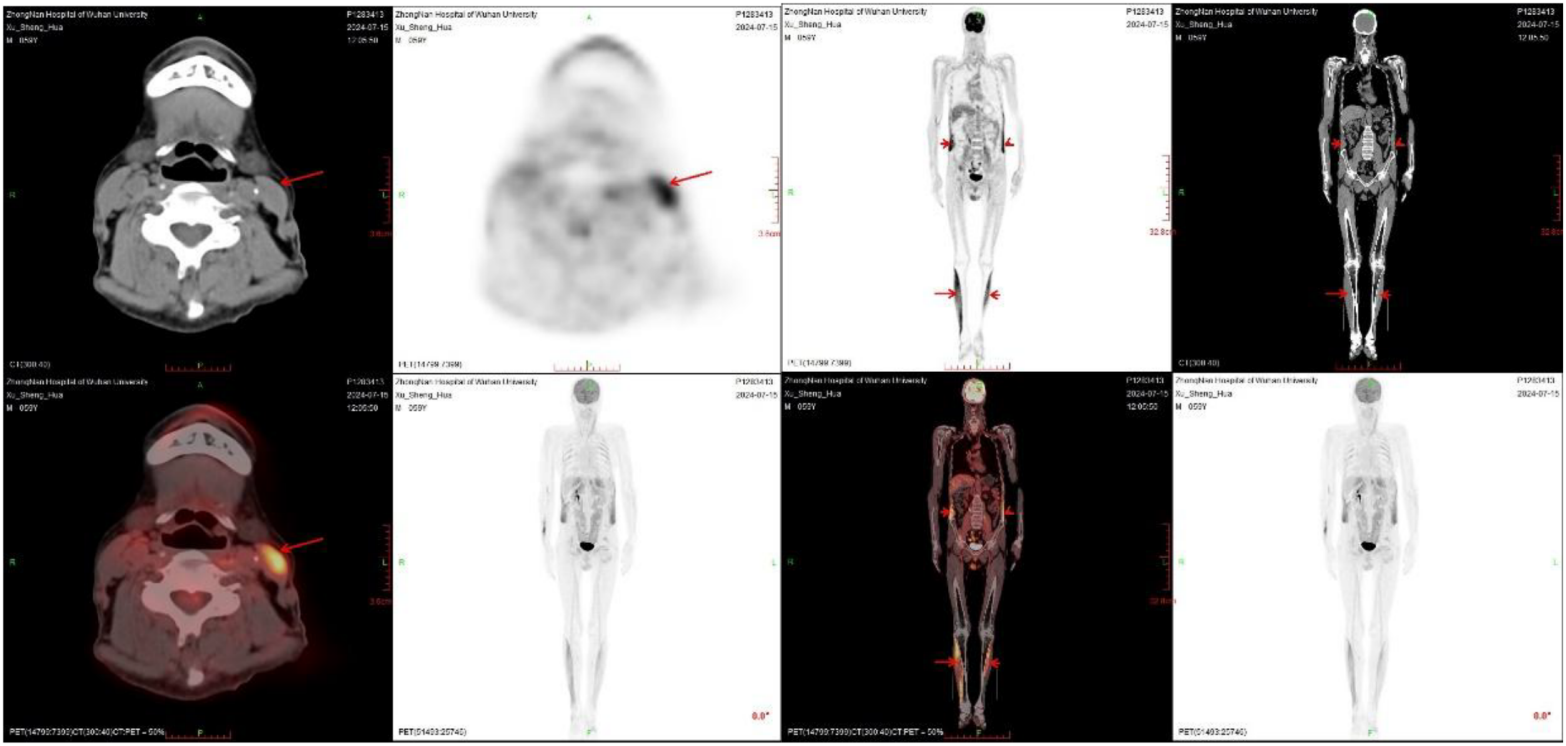
Whole-body PET–CT imaging in stiff-person syndrome. Symmetric increased ¹^8^F-fluorodeoxyglucose (FDG) uptake is seen in bilateral shoulder girdle muscles (standardized uptake value [SUV]_max_, 4.2), paraspinal muscles (SUV_max_, 3.6), and hip adductor muscles (SUV_max_, 3.9) (red arrows), reflecting sustained muscle contraction characteristic of stiff-person syndrome.

By June 2024, he developed generalized muscle rigidity and episodic spasms triggered by stress or bladder distention—features suggestive of stiff-person syndrome (SPS). Stiffness began in the trunk and extended to the limbs, producing episodes of “statue-like” immobility. Diagnostic support at the time included: (1) Clinical: Progressive axial and limb rigidity, spasms precipitated by emotional or visceral stimuli, immobilization typical of SPS. (2) Serological: Serum GAD65 antibody 1000 IU/mL (ref <30); CSF GAD65 antibody 100 IU/mL. (3) Imaging: Whole-body PET–CT showed symmetric FDG hypermetabolism in paraspinal (SUVmax 3.6), shoulder girdle (SUVmax 4.2), and hip adductor muscles (SUVmax 3.9), consistent with sustained muscle contraction (**Figure 3**). (4) Exclusion of mimics: Tests for neural paraneoplastic antibodies (including anti-NMDA and LGI1) were negative; brain MRI showed only mild white matter changes (Fazekas grade 1). Electromyography and muscle biopsy were not performed due to patient refusal. While these could provide additional supportive evidence, the diagnosis of SPS was firmly established based on the classic clinical phenotype, markedly elevated GAD65 antibodies in serum and CSF, and characteristic pattern of muscle hypermetabolism on PET-CT, after excluding alternative causes. He was treated with intravenous immunoglobulin (2 g/kg over 5 days) and clonazepam.

At 3-month follow-up (September 2024), Spasm frequency, assessed by patient diary, decreased from daily episodes to 1-2 episodes per week (approximately 85% reduction). The patient’s mobility improved from being wheelchair-bound to walking independently. Serum GAD65 antibody level had fallen by 54% (to 464 IU/mL), and CSF antibody level had dropped by 80%. Serum potassium normalized (4.1 mmol/L) without supplementation by the September 2024 follow-up, and urine pH decreased from 7.5 to 6.4 by January 2025. In January 2025, fasting plasma glucose was 14.3 mmol/L and glycated hemoglobin 11%, consistent with latent autoimmune diabetes in adults (LADA). Anti-insulin antibodies were present (165.5 IU/mL) but tests for antinuclear antibodies, anti–double-stranded DNA, anti-SSA/SSB, and other systemic autoimmune markers were negative, making coexistent connective tissue disease unlikely. The high glucose was managed with basal-bolus insulin therapy. GAD65 antibodies remained detectable (46.4 IU/mL). Thyroid ultrasonography showed diffuse parenchymal changes with a TI-RADS 3 nodule.

## Follow-up summary

From initial presentation in December 2023 to January 2025, the patient was followed for 13 months (Longitudinal laboratory and clinical data are summarized in **Table 1**). By September 2024, he regained mobility (no longer wheelchair-bound) and spasm frequency was markedly reduced. At last assessment (January 2025), he remained ambulant without assistance. Biochemical parameters were stable: serum potassium normal off supplementation; HbA1c 11% (insulin-managed); TSH improved but remained elevated, consistent with persisting subclinical hypothyroidism. He received one course of IVIG in June 2024 and continued clonazepam 1 mg/day for residual stiffness. No additional immunosuppressive therapy was required as of January 2025.

**Table 1:** Longitudinal clinical and laboratory parameters.

Parameter	December2023 (initial)	January2024 (prethymectomy)	Jun-24 (SPS diagnosis)	Sep-24 (3-month follow-up)	Jan-25 (last follow-up)
Serum potassium (mmol/L)	2.4	2.4	Not reported	Normal (no supplementation)	Normal
Serum bicarbonate (mmol/L)	—	9.7	—	—	—
Urine pH	—	7.5	—	6.7	6.4
TSH (μIU/mL)	—	15.3	—	—	17.9
Free T3(pg/ml)		2.46			2.78
Free T4(ng/dl)		0.864			0.92
2-h OGTT glucose (mmol/L)	—	9.3	—	—	28.8
Fasting glucose (mmol/L)	—	6.7	—	—	14.3
HbA1c (%)	—	5.8	—	—	11
Fasting C-peptide(ng/mL)		2.9			0.8
Serum GAD65 Ab (IU/mL)	—	—	1000	464 (↓54%)	46.4
CSF GAD65 Ab (IU/mL)	—	—	100	20 (↓80%)	—

OGTT, oral glucose tolerance test; Ab, antibody; CSF, cerebrospinal fluid; TSH, thyroid-stimulating hormone. ‘—’ indicates data not recorded or not applicable at that time point.

## Discussion

GAD65 autoantibodies appear to serve as the central pathogenic link in this patient’s multi-organ autoimmune syndrome, targeting GABAergic neurons, pancreatic β-cells, and renal tubules through conformational epitope exposure and cross-reactivity (5, 6, 10). GAD65, the enzyme responsible for synthesizing gamma-aminobutyric acid (GABA), is expressed in these diverse tissues. Its intrinsic structural flexibility—particularly in the pyridoxal phosphate (PLP)-deficient apo-state—exposes cryptic epitopes such as ²^60^PEVKEK²^65^, enhancing immunogenicity and enabling antibody cross-reactivity across organ systems (5, 6). This molecular plasticity distinguishes GAD65 from the more rigid isoform GAD67 and likely underlies its unique capacity to drive multi-system injury. While epitope mapping studies suggest structural homology as a basis for cross-reactivity (5, 6, 11), functional validation in cellular or animal models is required to confirm this pathogenic pathway.

In the nervous system, anti-GAD65 antibodies are thought to impair GABAergic inhibitory neurotransmission, leading to the muscle rigidity and spasms characteristic of stiff-person syndrome (SPS). The symmetric FDG hypermetabolism observed on PET–CT in paraspinal and proximal limb muscles provides functional imaging support for sustained motor neuron overactivity (13, 14). In the pancreas, endoplasmic reticulum stress in β-cells may promote aberrant palmitoylation and Golgi retention of GAD65, increasing its visibility to the immune system and facilitating T-cell–mediated destruction—culminating in latent autoimmune diabetes in adults (LADA) (12).

The association between GAD65 autoimmunity and dRTA is rare and its mechanism speculative. We hypothesize that cross-reactive T-cell clones—primed against thymoma-expressed GAD65—may traffic to the kidney. Local inflammation could disrupt H^+^-ATPase function in α-intercalated cells, analogous to tubulopathy in other autoimmune conditions (2). This proposed mechanism, while plausible given the epitope mapping data, remains to be proven by direct demonstration of GAD65-reactive cells or antibody deposition in renal tissue.

The progression to insulin-requiring diabetes in January 2025, despite reduced GAD65 antibody titers, highlights that clinical disease progression can continue even as humoral markers improve. This is consistent with the understanding that β-cell destruction in LADA is primarily T-cell mediated. The observed decline in fasting C-peptide (from 2.9 ng/mL pre-thymectomy to 0.8 ng/mL in Jan 2025) indicates substantial and likely irreversible loss of insulin-secretory capacity, which may not be halted by interventions primarily affecting antibody levels.

Subclinical hypothyroidism in this case may represent an extension of the autoimmune spectrum. Although anti-thyroid peroxidase (TPO) and anti-thyroglobulin (Tg) antibodies were not measured, which is a limitation, molecular mimicry between the GAD65 ²^60^PEVKEK²^65^ epitope and homologous sequences in TPO represents a potential mechanism for cross-reactivity (2).

A notable aspect of this case is the diagnosis of dRTA and endocrine abnormalities in December 2023, one month prior to the radiological identification of the thymoma in January 2024. This temporal sequence does not negate a causal role for the thymoma but rather reflects a well-documented clinical paradigm in thymoma-associated autoimmunity. Thymomas can disrupt central immune tolerance mechanisms (e.g., through AIRE downregulation and Treg impairment) long before the tumor becomes symptomatic or radiologically apparent (2, 15). The resulting autoreactive lymphocyte clones may then require a latent period before manifesting as overt organ injury, explaining why paraneoplastic syndromes often precede or coincide with tumor diagnosis (16). Furthermore, even after complete thymectomy, as in our case, these persistent autoreactive clones can continue to drive autoimmune injury, leading to new manifestations months or years later, a phenomenon reported in up to 30% of patients with thymoma and multiple autoimmune disorders (17). This pattern is analogous to myasthenia gravis, the most common paraneoplastic syndrome of thymoma, which frequently serves as the presenting symptom leading to the discovery of an occult tumor. Therefore, the sequence of events in our patient—autoimmune manifestations followed by tumor diagnosis and subsequent evolution of further autoimmunity post-thymectomy—fits within the established spectrum of thymoma-associated disease and reinforces the concept of the thymoma as the instigator and persistent amplifier of the autoimmune cascade. The underlying thymoma likely initiated this cascade through multiple, interrelated mechanisms of immune dysregulation (2, 9, 10). First, loss of AIRE expression in thymic medullary epithelial cells would impair intrathymic presentation of tissue-restricted antigens like GAD65, permitting the escape of autoreactive T cells (2, 9). Second, the tumor microenvironment showed diminished regulatory T-cell (Treg) function—a common feature in B2 thymomas—which may have failed to suppress emerging autoreactive clones (9). Third, ectopic expression of GAD65-like epitopes within the thymoma could have broken tolerance via molecular mimicry, generating cross-reactive B and T cells targeting neural, endocrine, and renal tissues (10). A limitation of our study is the lack of measurement of GAD65 antibody titers at the initial presentation in December 2023. Therefore, we cannot definitively establish whether high-titer antibodies preceded the thymoma diagnosis or surged following thymectomy/adjuvant therapy. However, the detection of extremely high titers (1000 IU/mL) at the time of SPS diagnosis in June 2024, coupled with the known persistence of autoreactive clones post-thymectomy, supports the interpretation that the thymoma was the primary source of immune dysregulation. The subsequent decline in antibodies following IVIG, paralleling clinical improvement, further underscores their pathogenic relevance.

Notably, clinical manifestations emerged sequentially over 18 months despite complete thymectomy, reflecting the persistence of autoreactive lymphocyte pools long after tumor removal—a phenomenon reported in up to 30% of thymoma patients who develop multiple autoimmune disorders (2, 10). The delayed onset may also reflect progressive epitope spreading, whereby initial immune responses against neuronal GAD65 broaden to structurally similar epitopes in kidney and thyroid, or differential tissue vulnerability based on local antigen density and MHC class II expression levels.

Recent structural studies further clarify GAD65’s pathogenic potential: in its apo-state, dynamic conformational changes expose immunodominant regions like ²^60^PEVKEK²^65^, whereas PLP-bound holoGAD65 remains stable and less immunogenic (5, 6). The pathogenic monoclonal antibody b96.11 has been shown to lock GAD65 dimers in an open conformation via CDRH3 interactions with this epitope, perpetuating autoantigen presentation and cross-reactive attack (5).

The clinical response to intravenous immunoglobulin (IVIG)—with a 53.6% reduction in serum and 80% decline in CSF GAD65 antibody levels—correlates with symptom improvement and suggests a direct pathogenic role for these antibodies (8). The rapid fall in CSF titers may reflect interruption of intrathecal antibody production, potentially mediated by mechanisms such as FcγRIIB-mediated inhibition of B-cell signaling or FcRn blockade, as suggested by prior studies (8).

This study, as a single-case report, has inherent limitations. First, the findings are based on the clinical experience of a single patient. While the proposed pathogenic cascade is compelling, it requires validation in larger cohorts of thymoma patients with multi-organ autoimmune manifestations. Second, the absence of key laboratory data at certain time points—such as GAD65 antibody titers at initial presentation and thyroid autoantibodies—and the lack of pathological confirmation from muscle or kidney biopsies due to patient refusal limit the depth of etiological verification. Specifically, the unavailability of renal histopathology or electromyography means we lack direct pathological evidence of immune-mediated tubulointerstitial nephritis or neurophysiological confirmation of continuous motor unit activity. Nevertheless, the diagnosis of dRTA was established on robust biochemical criteria after excluding other causes, and the diagnosis of SPS adhered to accepted clinical and serological criteria, supported by functional imaging. Third, the mechanistic explanations for cross-reactivity and treatment effects, though informed by the literature, remain hypothetical and necessitate direct experimental proof. Therefore, our conclusions, particularly regarding GAD65 as a “pan-autoantigen” and its antibody titer as a systemic biomarker, should be considered preliminary and suggestive. They highlight an important area for future collaborative research involving immunophenotyping, longitudinal antibody profiling, and functional studies.

**Figure 4:**
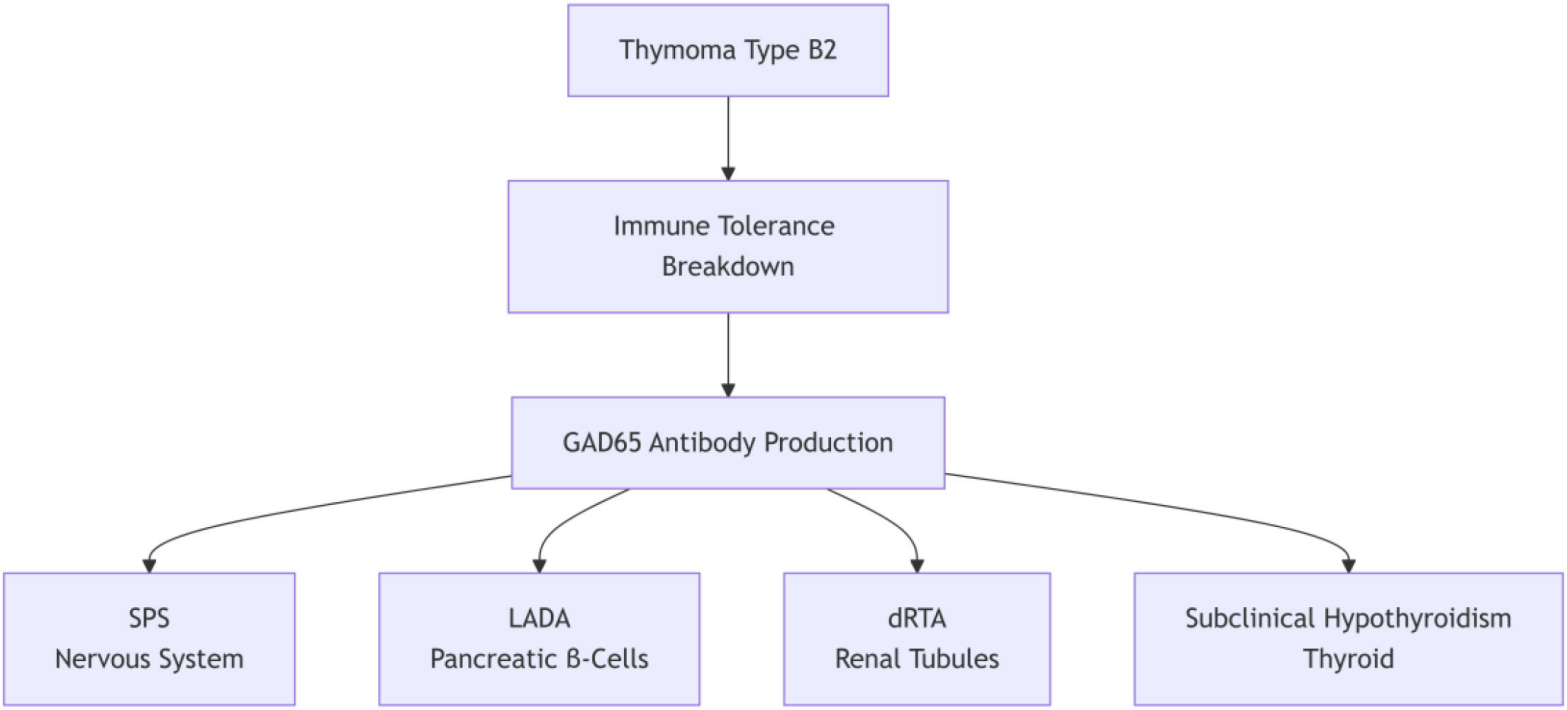
Pathogenic cascade of GAD65-mediated multi-organ autoimmunity.

In conclusion, this case unifies four autoimmune manifestations—SPS, dRTA, LADA, and thyroiditis—under a single pathogenic axis: thymoma-induced loss of tolerance to GAD65, followed by conformational epitope-driven cross-reactivity. The proposed cascade—Thymoma → Immune Dysregulation → GAD65 Autoantibodies → Multi-Organ Injury—underscores the need for multidisciplinary surveillance in thymoma patients with high-titer GAD65 antibodies and highlights GAD65 as a paradigmatic paraneoplastic pan-autoantigen (**Figure 4**).

## Diagnostic reasoning and challenges

The diagnosis of stiff-person syndrome (SPS) was based on the classic clinical presentation of axial rigidity and stimulus-sensitive spasms, supported by high-titer GAD65 antibodies in serum and CSF, and characteristic symmetric FDG hypermetabolism in paraspinal muscles on PET-CT. Distal renal tubular acidosis (dRTA) was confirmed by excluding other causes, demonstrating hyperchloremic metabolic acidosis with hypokalemia and inappropriately alkaline urine. The diabetes was classified as latent autoimmune diabetes in adults (LADA), based on adult onset, GAD65 antibody positivity, and progression from preserved insulin secretion to insulin dependency.

Key diagnostic challenges included the temporal sequence, where autoimmune symptoms preceded tumor detection by one month—a pattern consistent with paraneoplastic syndromes. Initial attribution of weakness solely to hypokalemia delayed recognition of SPS until rigidity and spasms emerged. Differential diagnoses for neurological symptoms ruled out myasthenia gravis (negative AChR antibodies), Isaac’s syndrome, and structural cord lesions. For metabolic acidosis, common causes like diarrhea and diabetic ketoacidosis were excluded, and primary aldosteronism was ruled out by imaging. While autoimmune polyendocrine syndromes were considered, the absence of adrenal insufficiency and other specific antibodies pointed toward a thymoma-driven paraneoplastic etiology rather than classic APS.

## Data Availability

The raw data supporting the conclusions of this article will be made available by the authors, without undue reservation.
